# One Dose versus Three Weekly Doses of Benzathine Penicillin G for Patients Co-Infected with HIV and Early Syphilis: A Multicenter, Prospective Observational Study

**DOI:** 10.1371/journal.pone.0109667

**Published:** 2014-10-06

**Authors:** Chia-Jui Yang, Nan-Yao Lee, Tun-Chieh Chen, Yu-Hui Lin, Shiou-Haur Liang, Po-Liang Lu, Wen-Chi Huang, Hung-Jen Tang, Chen-Hsiang Lee, Hsi-Hsun Lin, Yen-Hsu Chen, Wen-Chien Ko, Chien-Ching Hung

**Affiliations:** 1 Department of Internal Medicine, Far East Memorial Hospital, New Taipei City, Taiwan; 2 Department of Internal Medicine, National Cheng Kung University College of Medicine and Hospital, Tainan, Taiwan; 3 Department of Internal Medicine, Kaohsiung Medical University Hospital and College of Medicine, Kaohsiung Medical University, Kaohsiung, Taiwan; 4 Department of Internal Medicine, Kaohsiung Municipal Ta-Tung Hospital, Kaohsiung Medical University, Kaohsiung, Taiwan; 5 Department of Internal Medicine, Taichung Veterans General Hospital, Taichung, Taiwan; 6 Department of Internal Medicine, E-Da Hospital/I-Shou University, Kaohsiung, Taiwan; 7 Department of Internal Medicine, Kaohsiung Chang Gung Memorial Hospital, Chang Gung University College of Medicine, Kaohsiung, Taiwan; 8 Department of Internal Medicine, Chi-Mei Medical Center, Tainan, Taiwan; 9 Department of Internal Medicine, National Taiwan University Hospital and National Taiwan University College of Medicine, Taipei, Taiwan; 10 Department of Medical Research, China Medical University Hospital, Taichung, Taiwan; 11 China Medical University, Taichung, Taiwan; UNC Project-China, China

## Abstract

**Background:**

One dose of benzathine penicillin G (BPG) has been recommended for HIV-infected patients with early syphilis (primary, secondary, and early latent syphilis) in the sexually transmitted diseases treatment guidelines, but clinical data to support such a recommendation are limited.

**Methods:**

We prospectively observed the serological response to 1 or 3 weekly doses of BPG in HIV-infected adults who sought treatment of early syphilis at 8 hospitals around Taiwan. Rapid plasma reagin (RPR) titers were followed every 3–6 months after treatment. The serological response was defined as a 4-fold or greater decline in RPR titers at 12 months of treatment. The missing values were treated by following the last-observed-carried-forward principle. We hypothesized that 1 dose was non-inferior to 3 weekly doses of BPG with the non-inferiority margin for the difference of serological response set to 10%.

**Results:**

Between 2007 and 2012, 573 patients completed at least 12 months of follow-up: 295 (51.5%) receiving 1 dose of BPG (1-dose group) and 278 (48.5%) 3 doses (3-dose group). Overall, 198 patients (67.1%; 95% confidence interval [CI], 61.4–72.5%) in the 1-dose group achieved serological response at 12 months, as did 208 patients (74.8%; 95% CI, 69.3–79.8%) in the 3-dose group (one-sided 95% CI of the difference, 15.1%). In the multivariate analysis, secondary syphilis (adjusted odds ratio [AOR], 1.90; 95% CI 1.17–3.09), RPR titer ≥32 (AOR, 1.93; 95% CI, 1.38–2.69), and 3 doses of BPG (AOR, 1.68; 95% CI, 1.20–2.36) were independently associated with a serological response. The time to the first episode of treatment failure was 1184 (standard deviation [SD], 70.5) and 1436 (SD, 80.0) days for 1- and 3-dose group, respectively.

**Conclusions:**

Single-dose BPG resulted in a higher serological failure rate and shorter time to treatment failure than 3 weekly doses of BPG in the treatment of early syphilis in HIV-infected patients.

## Introduction

Syphilis, caused by *Treponema pallidum*, is an important sexually transmitted disease. Globally, there were an estimated 36.4 million cases of syphilis in adults between the ages of 15 and 49 years in 2008 [1]. The incidence of syphilis is increasing both in developed and less developed countries, especially among men who have sex with men (MSM) [2]–[5]. In the United Kingdom, the newly diagnosed cases of syphilis increased from 2,650 in 2010 to 2,915 in 2011 and 75% of the cases occurred in MSM [6]. In the United States, the case numbers reported to the Centers for Disease Control and Prevention (US CDC) increased from 13,774 in 2010 to 13,970 in 2011, an increase of 1.4%, and MSM were the major risk group, accounting for 83% and 72% in 2010 and 2011, respectively [7].

Appropriate treatment of early syphilis among HIV-infected patients has been controversial because clinical studies of a sufficient sample size have been lacking. In the previous version of treatment guidelines for syphilis by the US CDC in 2006 [8], an enhanced therapy with 3 weekly doses of benzathine penicillin G (BPG) (2.4 million units [MU]) was recommended by some experts for HIV-infected patients with early syphilis. Three weekly doses of BPG had been used for treatment of neurosyphilis in 1970s and several controlled trials had concluded clinical efficacy despite report of treatment failure in a few cases [9]. Therefore, enhanced therapy was recommended to provide protective effect against neurosyphilis in immunocompromised host [9]. However, a randomized control trial conducted before the introduction of combination antiretroviral therapy (cART) demonstrated no difference in treatment response between HIV-infected and HIV-uninfected patients treated with a single dose of BPG followed by a 10-day course of oral amoxicillin plus probenecid acid, although it is unclear if the use of additional depot penicillin would have resulted in better outcomes [10]. Therefore, a single dose of BPG for early syphilis has been recommended in the most recent guidelines in the UK, US and Europe [11]–[13]. However, dilemmas continue to exist among infectious diseases specialists as to the appropriate dose of BPG to be administered in the management of early syphilis in HIV-infected patients in the cART era [14].

Several studies have shown that HIV-infected patients with syphilis have higher failure rates after standard treatment, while other studies have not [15]–[18]. The discrepant results among these studies can be attributed to limitations of the published studies, such as a small sample size, lack of stratification for different stages of syphilis, different definitions of serological failure, high rates of patients who were lost to follow up, and enrollment of patients with low non-treponemal titers. In this study, we aimed to compare the serological response rates of early syphilis to 1 dose versus 3 weekly doses of BPG therapy and to identify the factors associated with a serological response among HIV-infected patients.

## Materials and Methods

### Study design and participants

This multicenter, prospective observational study was conducted between January 2007 and December 2012 at 8 major hospitals around Taiwan that are designated for HIV care by the Centers for Disease Control, Taiwan, where HIV care, including cART and monitoring of CD4 count and plasma HIV RNA load, is provided free-of-charge. According to the national guidelines for HIV care in Taiwan, non-treponemal serological tests for syphilis are recommended to be performed at least once yearly and on an as-needed basis as indicated by the clinical presentations and every 3 to 6 months over a period of 2 years for those who receive treatment for syphilis. The patients who received stable cART were usually followed as an outpatient every 3 months and monitoring of immunological and virological status was performed every 3 to 6 months.

HIV-infected patients were eligible for this observational study, if they were 20 years or older, had early syphilis (i.e. primary, secondary, or early latent), and had reactive rapid plasma reagin (RPR) titers of 1∶4 or greater and a reactive result of *Treponema pallidum* particle agglutination (TPPA) test (titer ≥1∶320), for which 1 or 3 weekly doses of BPG (2.4 MU, 1170 IU/mg, Biochemie Ges. M.B.H., Kundl, Australia) were administered intramuscularly. Patients with a prior history of syphilis who received treatment within 12 months before enrollment were excluded. In addition, patients were excluded if they were pregnant, received antibiotics such as penicillin, ceftriaxone, doxycycline, or macrolides for syphilis or other infections within the preceding 12 months or during follow-up, were lost to follow-up immediately after treatment, had a history of penicillin allergy, or were receiving immunosuppressants, immunomodulators, or chemotherapy. The study was approved by the Research Ethics Committee of each participating hospital and the written informed consent was waived.

### Diagnosis of syphilis

Primary syphilis was diagnosed when the patients presented with reactive serologies (RPR titers >1∶4 and reactive TPPA titers) and genital, anal, or oral ulceration (chancre); secondary syphilis was diagnosed when the patients presented with reactive serologies and cutaneous rashes, mucosal lesions, generalized lymphadenopathy, or other signs; and early latent syphilis was diagnosed when the patients presented with reactive serologies and documented negative RPR or 4-fold elevation of RPR titers with exposure to patients with syphilis within the preceding 12 months [11].

### Laboratory investigations

Serological tests for syphilis included the RPR test (BD Macro-VueTMRPR Card tests, USA) and *T. pallidum* particle agglutination test (FTI-SERODIA-TPPA. Fujirebio Taiwan Inc., Taoyuan, Taiwan). Plasma HIV RNA loads and CD4 lymphocyte counts were quantified with the use of the Cobas Amplicor HIV-1 Monitor™ Test, version 1.5, (Roche Diagnostics Corporation, Indianapolis, USA) and FACSFlow (Becton Dickinson), respectively.

### Treatment and follow-up

All patients with syphilis received 1 dose or 3 weekly doses of BPG. While treatment guidelines for syphilis evolved during the 5-year study period, the decision to give 1 or 3 doses of BPG depended on the assessment of the treating physicians, which could be influenced by the characteristics of each patient. Following treatment, follow-up of RPR titers every 3 to 6 months was recommended. A standardized case record form was used to collect information on demographic characteristics, a previous history of penicillin treatment for syphilis, stage of syphilis, RPR titers at baseline and during follow-up, dose of BPG administered, receipt of cART, and CD4 count and plasma HIV RNA load.

The primary endpoint of the study was achievement of serological response that was defined as a 4-fold or greater decline in RPR titers at the 12th month follow-up visit when compared with the baseline titers. The secondary endpoint included a 4-fold or greater decline in RPR titers at the 6th month follow-up visit when compared with the baseline titers; and the time to treatment failure, which was defined as the failure to achieve a 4-fold or greater decline in RPR titers; or a 4-fold or greater increase in RPR titers after achieving serological response during the 12 months of observation; or anytime when retreatment of syphilis was given during the 12 months of follow-up.

### Statistical analysis

All statistical analyses were performed using SPSS version 18.0 (SPSS, Chicago, IL) and Stata software, version 10 (StataCorp, College Station, TX, USA). All patients who received at least 1 dose of BPG were included for analyses, for which the last-observed-carried-forward principle was adopted to deal with missing data at the 6th and 12th month of follow-up. Each patient was followed at least for twice during the 12-month study period. Categorical variables were compared by Fisher’s exact test or Chi-square test. Non-categorical variables were compared by Mann-Whitney U test. Factors with a *P* value ≤0.2 or biological significance were included in the multivariate analysis. Binary logistic regression analysis was used to determine the factors associated with serological responses at the time of 12 months of follow-up. We fit the data using mixed effect model in which the covariate structure of “hospitals” was considered in the model. Thus the factor “hospital” was treated as a random effect and was put into mixed effect binary logistic regression analysis. All comparisons were 2-tailed and a *P* value <0.05 was considered to be significant. We hypothesized that 1-dose BPG regimen was non-inferior to the 3-dose regimen in the HIV-infected patients with early syphilis. The non-inferiority in terms of serological response rate following 1-dose BPG therapy to 3-dose therapy would be concluded if the lower boundary of the two-sided 95% confidence interval (CI) (one-sided α = 0.025) for the difference in the serological response rate between the two groups was at least −0·1 (that is, the non-inferiority margin was set to 10%). With the estimated serological response rate of 75% for the 3-dose BPG group, it was estimated that 232 patients were needed for each group to confirm the non-inferiority of 1-dose to 3-dose regimen with a power of 80%. Time to treatment failure was assessed by Kaplan-Meier plots and Cox proportional hazards regression analysis was used to estimate the hazard ratios (HR) for treatment failure between the two groups of patients. All patients were followed until 30 June, 2013, or the date when the patients received the diagnosis of recurrent syphilis or were lost to follow-up, or death, whichever occurred first.

## Results

**Figure 1:**
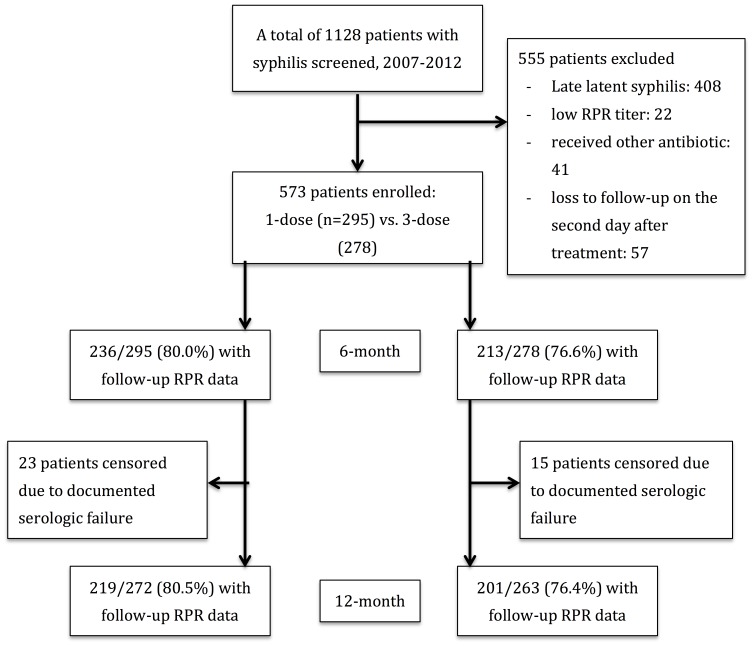
Study flow showing patient recruitment and patients with available rapid plasma reagin (RPR) data during the 6 and 12 months of follow-up.

During the 5-year study period, 1128 HIV-infected patients with syphilis sought HIV care and treatment of syphilis at the 8 participating hospitals, and 555 (49.2%) patients were excluded due to late latent syphilis stage (n = 408), concurrent use of other antibiotics (n = 41), loss to follow-up on the second day after treatment (n = 57; 26 for 1-dose and 31 for 3-dose group), low RPR titers (<1∶4) (n = 22), and without confirmed HIV infection (n = 8) (Figure 1). A total of 573 HIV-infected patients, including 295 patients receiving 1 dose and 278 patients receiving 3 doses of BPG, were enrolled. During the study period, the proportion of patients receiving 1 dose of BPG increased after 2010, when the updated treatment guidelines of US CDC in 2010 recommended 1 dose of BPG for early syphilis in HIV-infected patients (Figure S1).

Baseline characteristics of the two groups of patients are shown in Table 1. Most patients were MSM. There were no statistically significant differences between the two groups of patients in terms of age, sex, gender, the stage of syphilis, RPR titer, a prior history of syphilis, CD4 count, plasma HIV RNA load, and receipt of cART (Table 1). Of note, more than half (57.8%) of the patients had secondary syphilis at enrollment, 64% had baseline CD4 cell counts of more than 350 cells/µl, and 35.4% had a prior history of syphilis. While missing values during follow-up were dealt using the last-observed-carried-forward principle, no statistically significant differences were observed for most of the clinical characteristics assessed between the patients with missing values and those without missing values at the 6th and 12th months of follow-up (Tables S1 and S2).

**Table 1:** Clinical characteristics of HIV-infected patients with early syphilis receiving 1 dose or 3 weekly doses of benzathine penicillin G.

	All patients (n = 573)	1-dose (n = 295)	3-dose (n = 278)	*P-*value
Age, mean (SD), years	33.1 (7.8)	32.8 (7.9)	33.5 (7.8)	0.27
Risk for HIV transmission, n (%)				
MSM	539 (94.1)	284 (96.3)	255 (91.7)	NA
Heterosexuals	23 (4.0)	8 (2.7)	15 (5.4)	NA
Others	11 (1.9)	3 (1.0)	8 (2.9)	NA
Syphilis stage, n (%)				
Primary	51 (8.9)	28 (9.5)	23 (8.3)	0.66
Secondary	331 (57.8)	173 (58.6)	158 (56.8)	0.67
Early latent	191 (33.3)	94 (31.9)	97 (34.9)	0.48
RPR titer, median [IQR]	1∶64 (32, 128)	1∶64 (32, 128)	1∶64 (32, 128)	0.61
CD4 count, mean (SD), cells/µl	457 (244)	462 (239)	452 (249)	0.62
CD4≤200, n (%)	71 (12.4)	33 (11.2)	38 (13.7)	0.38
CD4≤350, n (%)	207 (36.1)	103 (34.9)	104 (37.4)	0.54
PVL, mean (SD), log_10_ copies/ml	3.04 (1.49)	3.02 (1.51)	3.06 (1.48)	0.73
PVL <400 copies/ml, n (%)	305 (53.2)	156 (52.9)	149 (53.6)	0.87
Prior history of syphilis n (%)	203 (35.4)	103 (34.9)	100 (36.0)	0.79
CART, n (%)	362 (63.2)	194 (65.8)	168 (60.4)	0.26

**Abbreviations:** CART, combination antiretroviral therapy; IQR, interquartile range; MSM, men who have sex with men; PVL, plasma HIV RNA load; RPR, rapid plasma reagin; SD, standard deviation.

**Figure 2:**
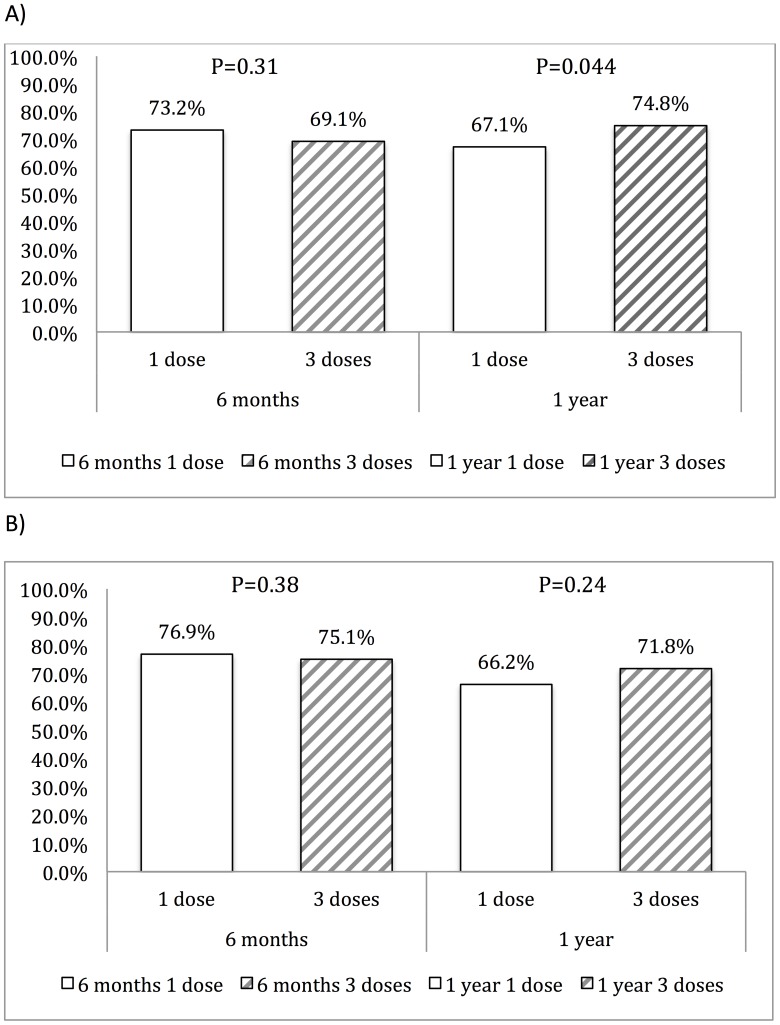
Serological response rates in the 1-dose and 3-dose benzathine penicillin G groups at 6 months and 12 months of follow up. A). Last-observed-carried-forward analysis; B). Per-protocol analysis.

The serological response rate at 12 months of BPG treatment was 67.1% (95% CI, 61.4–72.5) for the 1-dose group and 74.8% (95% CI, 69.3–79.8) for the 3-dose group (*P* = 0.044) in the last-observed-carried-forward population (Figure 2A). In the per-protocol population, the serological response rate was 66.2% (95% CI, 59.6–72.4) and 71.8% (95% CI, 64.7–78.2), respectively, for the 1-dose and 3-dose group (*P* = 0.24) (Figure 2B). The difference of serological response rates between 3-dose and 1-dose BPG group was 7.7% (95% CI, 0.3–15.1; *P* = 0.041) in the last-observed-carried-forward population, with the one-sided 95% CI of effectiveness difference between the 3-dose and 1-dose group exceeding the predefined 10% non-inferiority margin.

In the analysis of primary endpoint, the factors associated with a serological response in univariate and multivariate analysis are shown in Table 2. In univariate analysis, treatment with 3 weekly doses of BPG and secondary syphilis were associated with a higher serological response rate, while early latent syphilis was associated with a lower serological response rate. Receipt of cART, CD4 count (either <200, between 200 and 350 or >350 cells/µl), or plasma HIV RNA load was not statistically significantly associated with serological response, although a trend toward a higher serological response rate was observed in patients with CD4 counts >350 cells/µl (*P* = 0.084). In the multivariate logistic regression analysis, we found that secondary syphilis (adjusted odds ratio [AOR], 1.90; 95%CI, 1.17–3.09), RPR titers ≥32 (AOR, 1.93; 95%CI, 1.38–2.69), and 3-dose BPG (AOR, 1.68; 95%CI, 1.20–2.36) were independently associated with a serological response (Table 2).

**Table 2:** Factors associated with serological response in the patients who received 1 dose or 3 doses of benzathine penicillin G at 12 months of follow-up in univariate and multivariate analysis.

	Univariate analysis			Multvariate analysis		
	Responders(n = 406)	non-responders(n = 167)	*P*-value	Adjusted oddsratio	95% confidenceinterval	*P*-value
Age, mean (SD), years	33.1 (8.0)	33.2 (7.3)	0.89	1.01	0.99–1.03	0.55
Risk, n (%)						
MSM	386 (95.1)	153 (91.6)	0.118	1.67	0.82–2.89	0.19
non–MSM	20 (4.9)	14 (8.4)		1	–	–
Syphilis stage, n (%)						
Primary	34 (8.4)	17 (10.2)	0.52	1	–	–
Secondary	249 (61.6)	82 (49.1)	0.005	1.90	1.17–3.09	0.01
Early latent	123 (30.3)	68 (40.7)	0.014	1.04	0.70–1.56	0.84
RPR titer, median (IQR)	1∶64 (32, 128)	1∶64 (32, 128)				
RPR titer 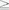 1∶32	353 (86.9)	125 (74.9)	0.001	1.93	1.38–2.69	<0.001
CD4 count, mean (SD), cells/µl	460 (242)	449 (249)	0.61			
CD4 ≦200, n (%)	47 (11.6)	24 (14.3)	0.49	1	–	–
200<CD4 ≦350, n (%)	89 (21.9)	47 (28.1)	0.16	1.05	0.54–2.07	0.88
CD4>350, n (%)	270 (66.5)	96 (57.5)	0.084	1.51	0.69–3.51	0.30
PVL, mean (SD), log10 copies/ml	3.07 (1.51)	2.99 (1.47)	0.53			
PVL <400 copies/ml, n (%)	192 (47.3)	76 (45.5)	0.58	0.97	0.78–1.21	0.79
Prior history of syphilis, n (%)	142 (35.0)	61 (36.5)	0.77	0.98	0.79–1.21	0.82
CART, n (%)	251 (61.8)	111 (66.5)	0.29	0.75	0.47–1.20	0.23
3 doses of penicillin, n (%)	208 (51.2)	70 (41.9)	0.04	1.68	1.20–2.36	0.002

**Abbreviations:** CART, combination antiretroviral therapy; IQR, interquatile range; PVL, plasma HIV RNA load; RPR, rapid plasma reagin; SD, standard deviation.

In the two groups of patients, 88.1% and 86.0% in the 1-dose group and the 3-dose group, respectively, ever achieved a 4-fold or greater decline in RPR titers after treatment (data not shown) (*P* = 0.46). There were no statistically significant differences in the overall serological response rates before and after the change of treatment guidelines in 2010 [11]; however, a lower serological response rate to the 1-dose BPG was observed after 2011 (Figure S2).

**Figure 3:**
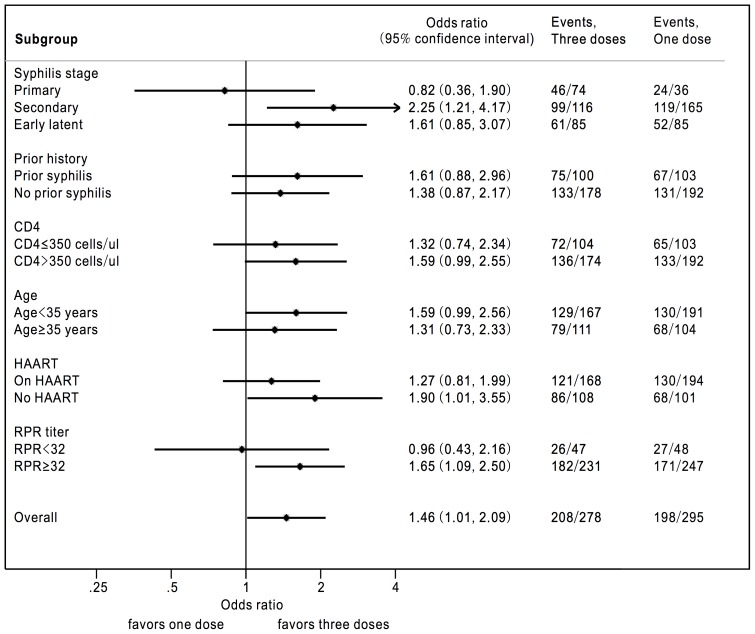
Forest plot showing the serological response rates in different subgroups of patients receiving 1 or 3 doses of benzathine penicillin G at 12 months of follow-up.

The serological response rates in different subgroups at 12 months of follow-up are shown in the forest plot (Figure 3). The subgroup analysis demonstrated the consistency with overall serological response favoring 3 weekly doses of BPG except for the subgroups of patients who presented with primary syphilis and RPR titers <32.

We also evaluated the serologic response at 6 months of follow-up with univariate analysis and mixed effect binary logistic regression (Table S3 and Figure S4). No significant difference was observed of the serologic response rate between the 1-dose group and 3-dose group while RPR titers ≧32 and CD4 counts >350 cells/µl were found to be associated with serologic response after treatment.

**Figure 4:**
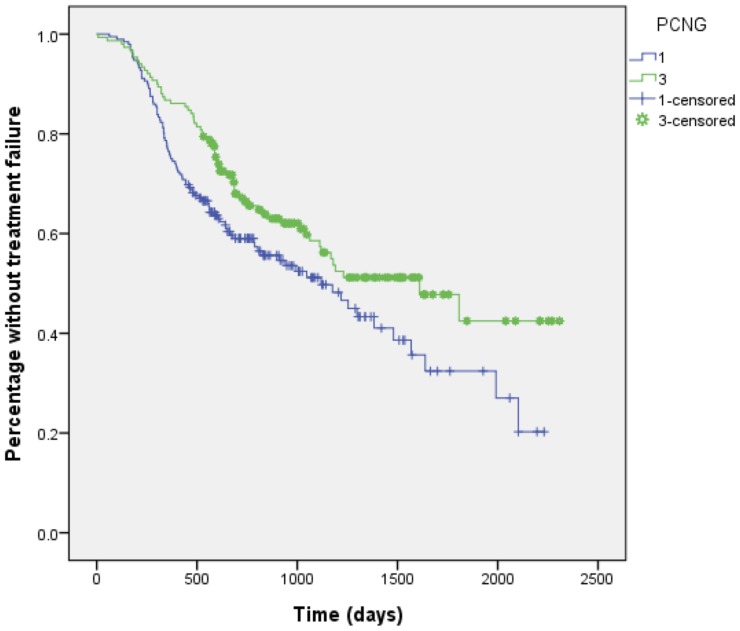
Kaplan-Meier plot showing the time to treatment failure to 1- or 3-dose benzathine penicillin G regimen (Log rank test, *P* = 0.026).

During the study period, the mean observation duration was 1102 days (standard deviation [SD], 488) for the 1-dose group and 1265 days (SD, 483) for the 3-dose group. Overall, treatment failure developed in 146 (49.5%) and 120 (43.2%) patients in the 1-dose and 3-dose group (p = 0.13), respectively, during the follow-up; none developed clinical manifestations suggestive of neurosyphilis, however. The Kaplan-Meier survival plot to depict the time to treatment failure for the two groups of patients is illustrated in Figure 4. The mean time to treatment failure was significantly longer for the 3-dose group than the 1-dose group (1436 [SD, 80.0] vs 1184.days [SD, 70.5]; log-rank test, *P* = 0.026).

In each group of patients who developed treatment failure during the follow-up, more patients in the 1-dose group than 3-dose group experienced an increase of RPR titers by 4-fold or greater at 6 months of follow-up (16.7% [13/78] vs 3.5% [3/85], *P* = 0.007) as well as at 12 months of follow-up (70.0% [64/97] vs 47.1% [33/70], *P* = 0.018) (Figure S3). During the follow-up period, there were 5 patients with documented reinfections with primary syphilis, 17 with secondary syphilis and 6 with both primary and secondary syphilis in the 1-dose group (28.7%), while there were only 2 patients with reinfections with primary syphilis and 5 secondary syphilis in the 3-dose group (10%). In addition, 6 and 2 patients in the 1-dose and the 3-doses BPG group, respectively, received retreatment because of a delayed serological response.

## Discussion

In this multicenter, prospective observational study, we demonstrated that 1-dose BPG was inferior to the 3-dose regimen in achieving a 4-fold or greater decline in RPR titers at 12 months of follow-up since a higher failure rate and a shorter time to treatment failure were noted in the 1-dose group. The better serological response was associated with higher RPR titers (RPR≥32) at baseline, secondary syphilis, and 3-dose BPG therapy. Although not statistically significant, we found a trend that a higher serologic response rate was associated with higher CD4 cell counts.

In HIV-infected patients with syphilis, the serological response rates to BPG ranged from 70.4% to 91.8% in previous retrospective studies that were conducted in the cART era [15]–[18]. In our study, the serological response rate at 12 months in the 1-dose and 3-dose group was 67.1% and 74.8%, respectively, while the overall serological response rate was 70.9%. In contrast to other studies that included a higher percentage of female patients or a lower proportion of MSM [15]–[17], our study population was mainly composed of subjects who were MSM, a population that have been reported to have a higher rate of syphilis reinfection [19], which may have contributed to the lower serological response rate observed in our study. In the retrospective study by Farhi et al, in which 95% of the population were MSM and 53% presented with secondary syphilis, the serologic response rate is higher (91.8%) than ours [18]. However, more than one fourth of the patients were excluded from the final analysis because of inadequate information; furthermore, a relatively higher preserved immune status was observed in the study population [18]. The association between lower CD4 cell counts (<200 cells/µl) and a higher serologic failure rate has been reported in a previous study [20], which was also observed in our study (Table 2).

In this study, we failed to demonstrate that the serological response of the 1-dose group was non-inferior to that of the 3-dose group during the 12-month follow-up. The subgroup analysis demonstrated the consistency with overall serological response favoring 3 weekly doses of BPG except for the subgroups of patients who presented with primary syphilis and RPR titers <32 (Figure 3). The results of a better serological response for the 3-dose group could be multifactorial, though more investigations are warranted. In our study, the interval between treatment and treatment failure for the 3-dose group was longer than that for the 1-dose group. The receipt of 3 weekly doses of BPG could theoretically maintain a longer duration of therapeutic level of BPG than a single dose, which suggests that 3-doses BPG could have a longer protective effect [21]. However, no cases of early treatment failure were noted within the first 3 months of treatment with either 1 dose or 3 doses of BPG; furthermore, the proportions of the patients ever achieving a 4-fold or greater decline of RPR titers were similar between the two groups of patients. Therefore, the difference of serological response rates between these two groups could be attributed to other reasons. One retrospective study with a large sample size concluded that no difference of serologic failure rates between 1-dose and 3-dose BPG group in treating early syphilis of HIV-infected patients [22]. However, extremely unequal sample sizes of 1-dose (n = 47) and 3-dose BPG (n = 434) groups with potential bias might make the conclusion less convincing. In addition, the follow-up of RPR titers within the study period was not mentioned in detail, which might result in underestimation of the rate of reinfection.

In our study, the patients who failed to achieve a serological response often had a 4-fold increase of RPR titer after ever achieving a serological response during the 12-month follow-up period or experienced primary or secondary syphilis suggesting reinfection with syphilis, which was more frequently observed in the patients who received 1 dose of BPG than those who received 3 weekly doses. Given the long interval between treatment administration and treatment failure, our findings of a lower response rate to 1 dose BPG therapy than to 3 weekly doses may result from higher rates of reinfection with syphilis, suggesting that more intensive safe-sex counseling is needed to be implemented in this high-risk population.

The strengths of our study include that it is a prospective observational study with a large sample size and the two groups of patients had similar baseline characteristics (Table 1), including prior syphilis; only patients with early syphilis were enrolled and those with low RPR titers (<4) were excluded; and all of the treatment responses were evaluated at 6 and 12 months of follow-up [11]. Moreover, the patients who had ever received the other antibiotics, such as doxycycline, azithromycin, amoxicillin or ceftriaxone, were excluded.

There are several limitations of our study, however. First of all, this is not a randomized clinical trial and the decision to give 1 dose or 3 doses of BPG depended on the assessment of physicians who might prefer to give the more enhanced, 3 doses of BPG to patients who were considered at a higher risk for recurrent syphilis or treatment failure such as a previous history of syphilis, higher RPR titers, early latent syphilis, or lower CD4 counts, higher plasma HIV RNA loads, a history of prior opportunistic infections and more advanced HIV disease. However, there were no statistically significant differences in the baseline characteristics when treatment for syphilis was administered between the two study groups in our study. Second, nearly one fourth of the patients had missing RPR titers at 6 and 12 months following treatment. However, if we analyze the patients with available 6-month and 12-month RPR titers, the response rate is still nearly the same between 1-dose and 3-doses BPG in 12-month (Figure 2B). Third, the validity of using serologic response as a true testament of cure remains to be investigated [23]. Because we have no clinical standard to determine bacteriologic cure, in the case of syphilis we used a 4-fold or greater decline of RPR titers as an evidence of cure as suggested by the treatment guidelines [11]. However, it’s difficult to differentiate relapse from reinfection based on increased RPR titers following completion of treatment. More cases of primary and/or secondary syphilis were seen in the 1-dose group than 3-dose group during the follow-up (28.9% vs. 10%, *P* = 0.004). Therefore, we believe that more episodes of reinfection could contribute to the higher failure rate in the 1-dose group. Fourth, 94% of the patients in our study were MSM, and therefore our results may not be generalizable to other risk groups. Last, we did not perform cerebrospinal fluid study in patients with higher RPR titers or lower CD4 counts, as suggested by previous investigators [11]. However, no neurologic symptoms of all the patients had been observed during the follow-up period (data not shown).

In summary, we failed to demonstrate the non-inferiority of 1 dose of BPG to 3 weekly doses of BPG for treatment of early syphilis in HIV-infected patients. A substantial rate of treatment failure due to reinfection in both groups suggests that counseling for risk behavior modification should be integral component of management of HIV-infected patients with early syphilis.

## Supporting Information

Figure S1 **Trends of the proportions of the patients receiving 1 dose or 3 weekly doses of benzathine penicillin G between 2007 and 2012 before and after revision of Sexually Transmitted Diseases Treatment Guidelines by the US Centers for Disease Control and Prevention in 2010.** The number inside the bar indicates the number of the patients receiving 1 dose (stippled) or 3 weekly doses (gray) of benzathine penicillin G.
(TIFF)

Figure S2 **The serological response rates of 1 or 3 doses of benzathine penicillin G therapy before and after the revision of Sexually Transmitted Diseases Treatment Guidelines in 2010.**
(TIFF)

Figure S3 **The causes of treatment failure for the two groups of patients at 12 months of treatment. The numbers inside the bars indicate the cases of treatment failure.**
(TIFF)

Figure S4 **Forest plot showing the serological response rates in different subgroups of patients receiving 1 or 3 doses of benzathine penicillin G at 6 months of follow-up.**
(TIFF)

Table S1 **Comparisons of clinical characteristics of patients with missing rapid plasma reagin values and those without missing values at 6 months of follow-up.**
(DOCX)

Table S2 **Comparisons of clinical characteristics of patients with missing rapid plasma reagin values and those without missing values at 12 months of follow-up.**
(DOCX)

Table S3 **Factors associated with serological response in the patients who received 1 dose or 3 doses of benzathine penicillin G at 6 months of follow-up in univariate and multivariate analysis.**
(DOC)
