# Supplementary figures and images for: One Dose versus Three Weekly Doses of Benzathine Penicillin G for Patients Co-Infected with HIV and Early Syphilis: A Multicenter, Prospective Observational Study
Source: PLoS One. 2014 Oct 6;9(10):e109667. doi: 10.1371/journal.pone.0109667 (PMC4186862; doi:10.1371/journal.pone.0109667)

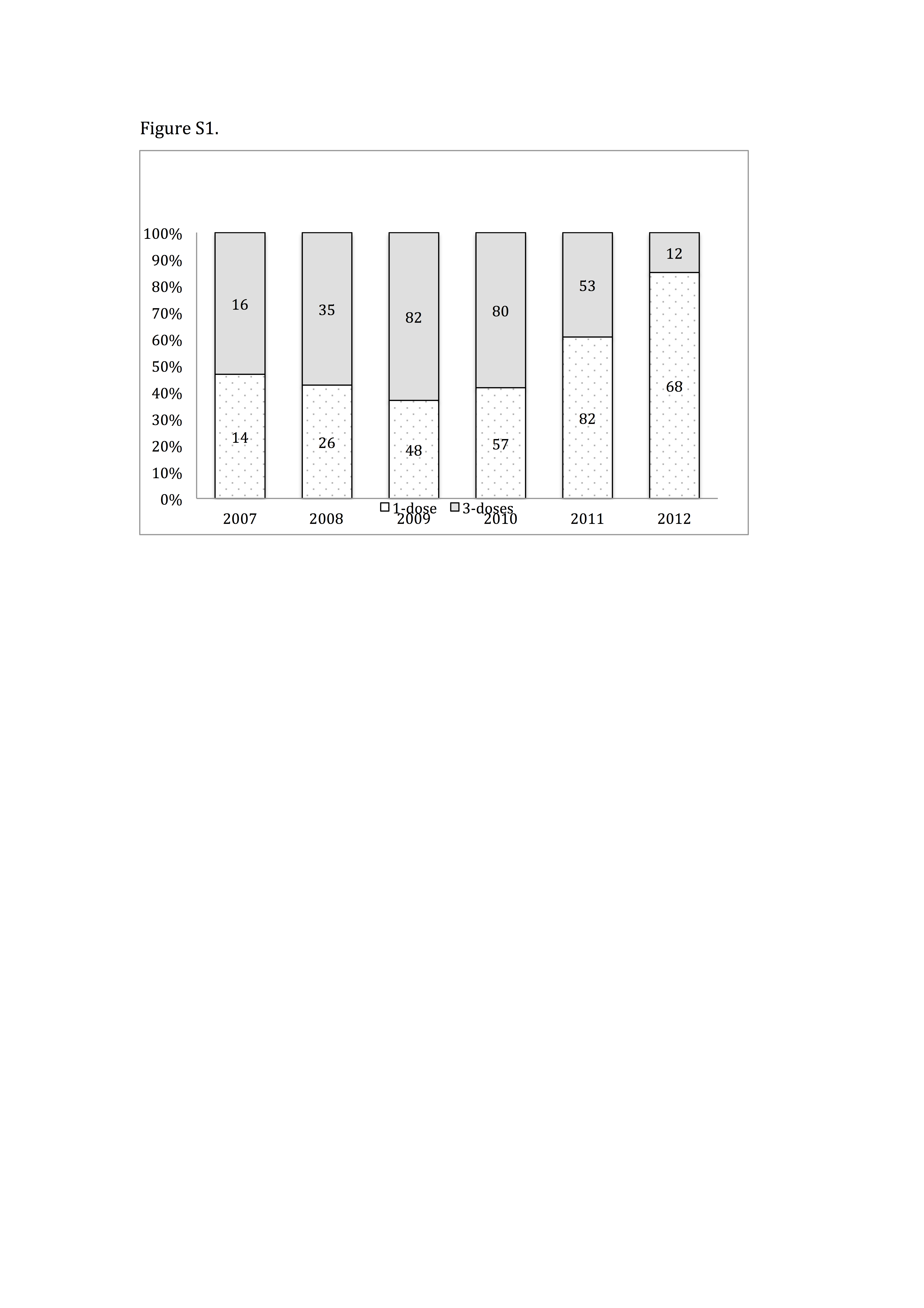

Supplement: Figure S1 — Trends of the proportions of the patients receiving 1 dose or 3 weekly doses of benzathine penicillin G between 2007 and 2012 before and after revision of Sexually Transmitted Diseases Treatment Guidelines by the US Centers for Disease Control and Prevention in 2010. The number inside the bar indicates the number of the patients receiving 1 dose (stippled) or 3 weekly doses (gray) of benzathine penicillin G. (TIFF) [file pone.0109667.s001.tiff]

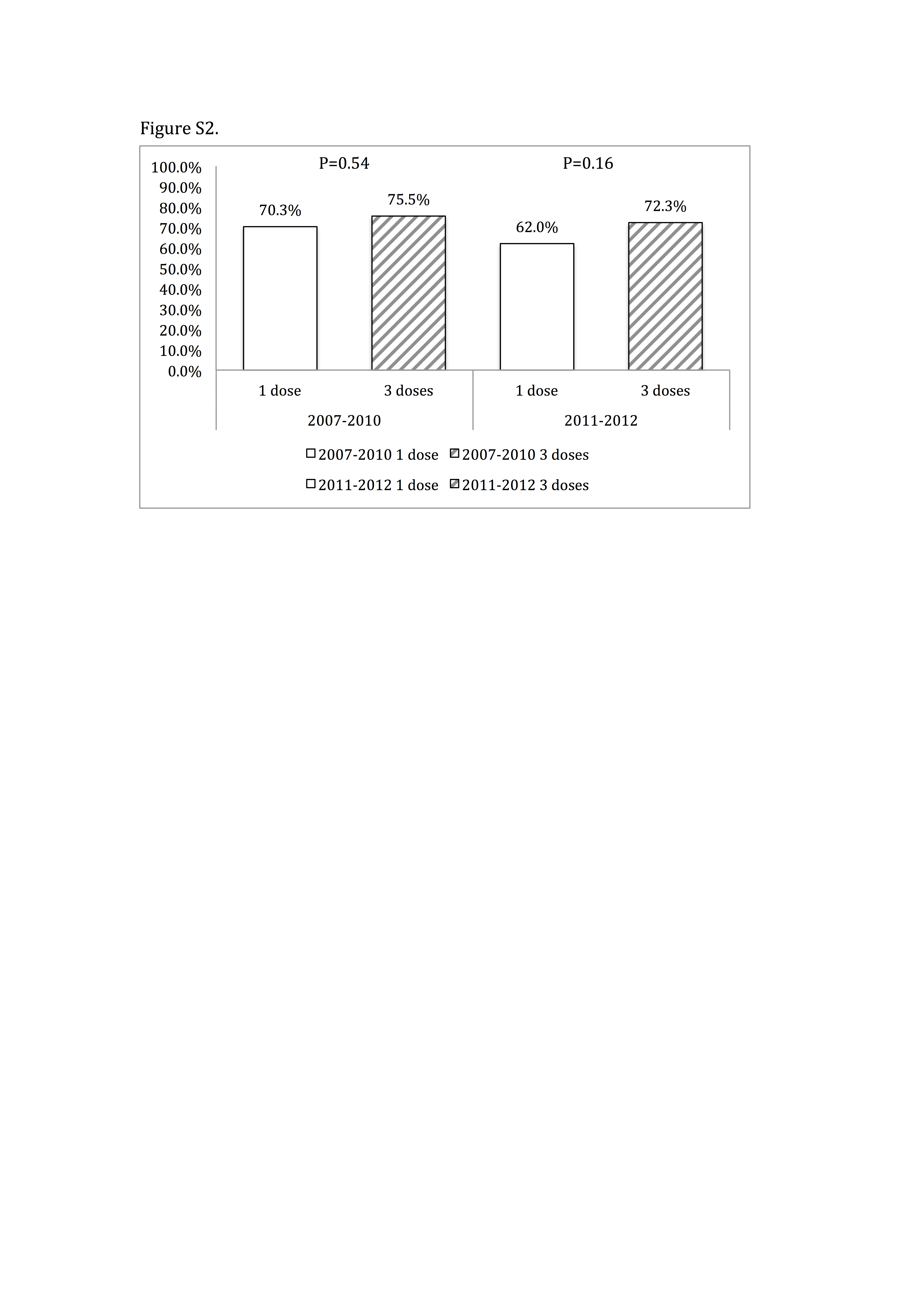

Supplement: Figure S2 — The serological response rates of 1 or 3 doses of benzathine penicillin G therapy before and after the revision of Sexually Transmitted Diseases Treatment Guidelines in 2010. (TIFF) [file pone.0109667.s002.tiff]

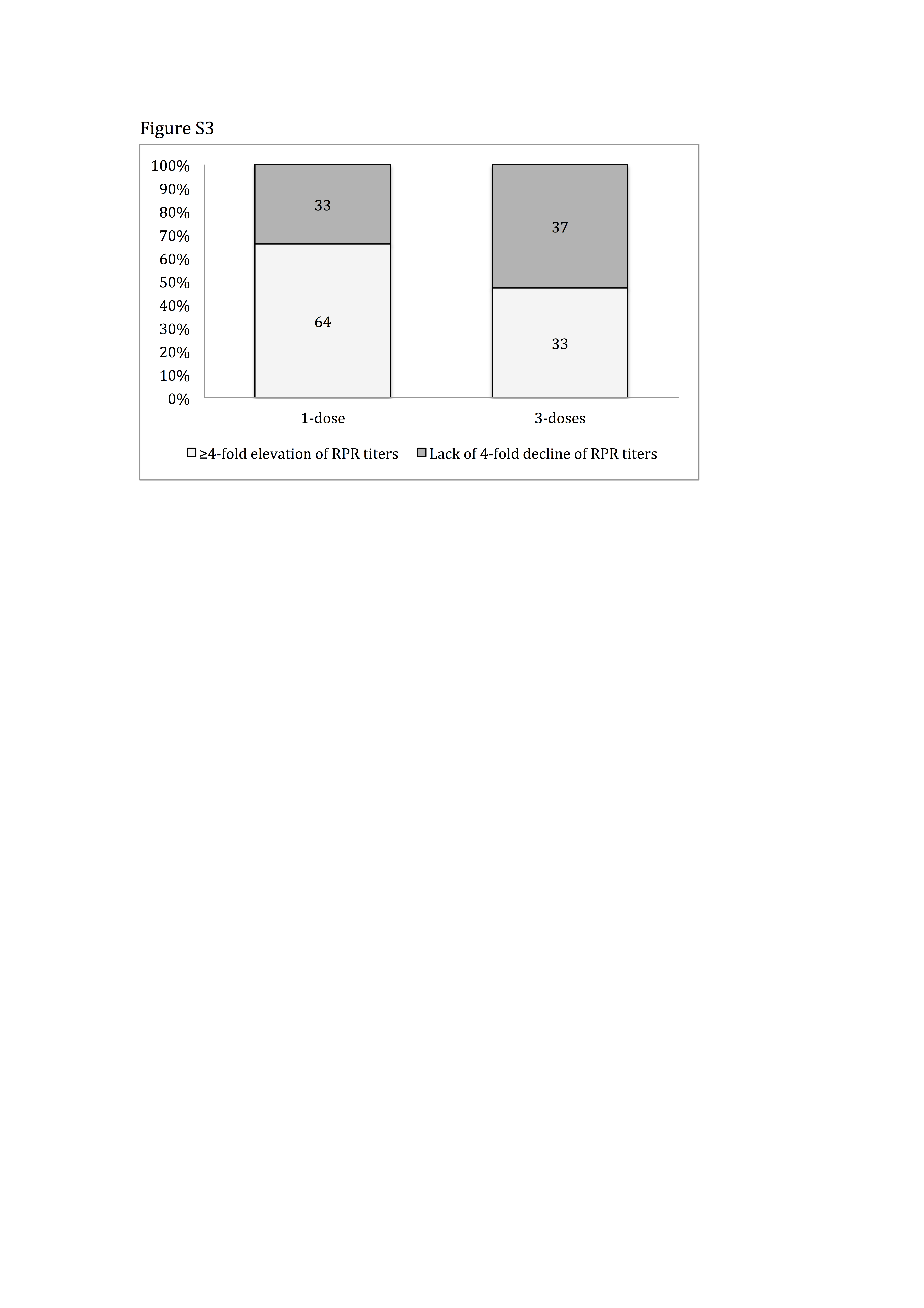

Supplement: Figure S3 — The causes of treatment failure for the two groups of patients at 12 months of treatment. The numbers inside the bars indicate the cases of treatment failure. (TIFF) [file pone.0109667.s003.tiff]

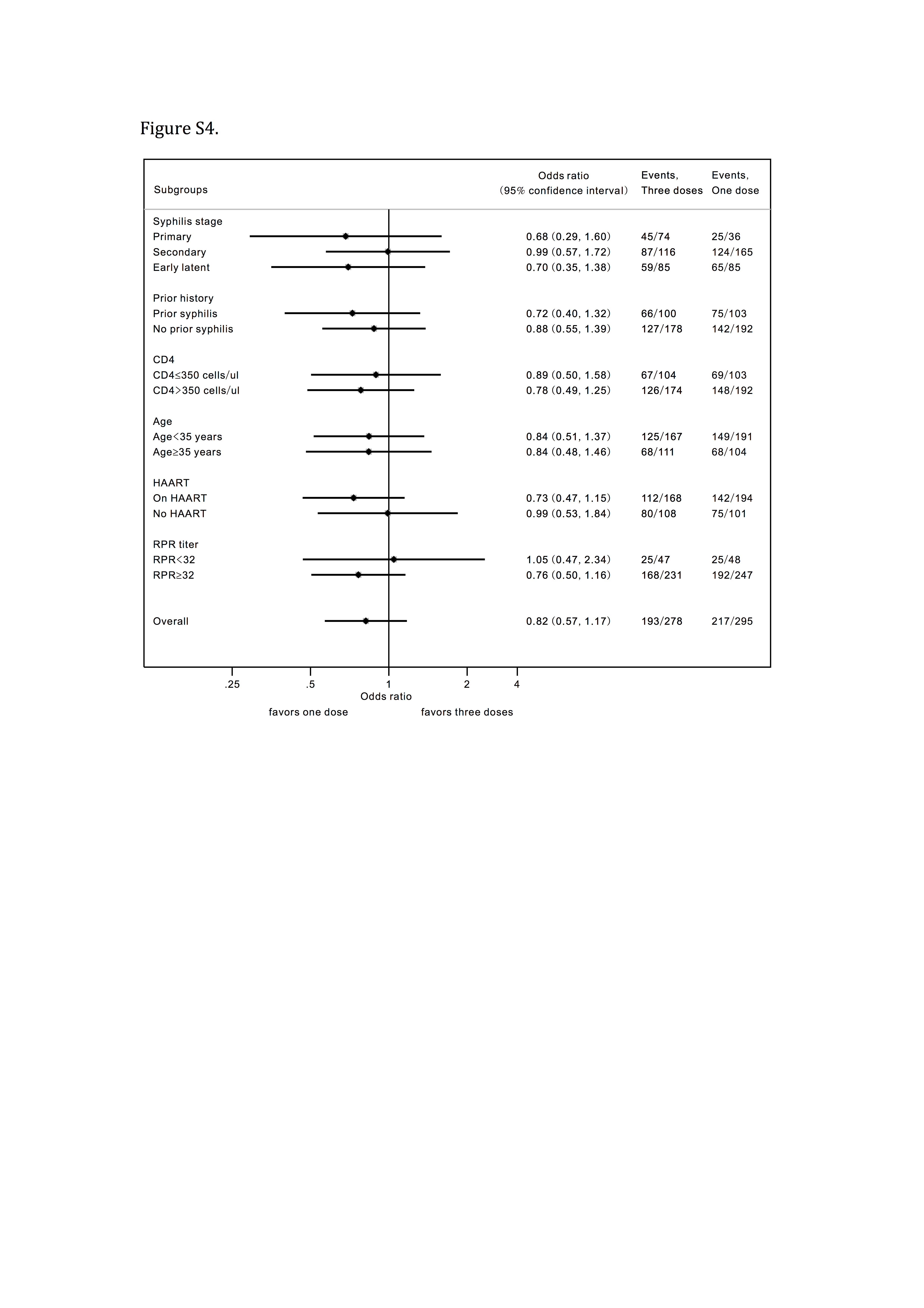

Supplement: Figure S4 — Forest plot showing the serological response rates in different subgroups of patients receiving 1 or 3 doses of benzathine penicillin G at 6 months of follow-up. (TIFF) [file pone.0109667.s004.tiff]
