# Supplementary material for: One Dose versus Three Weekly Doses of Benzathine Penicillin G for Patients Co-Infected with HIV and Early Syphilis: A Multicenter, Prospective Observational Study
Source: PLoS One. 2014 Oct 6;9(10):e109667. doi: 10.1371/journal.pone.0109667 (PMC4186862; doi:10.1371/journal.pone.0109667)
Supplement: Table S1 — Comparisons of clinical characteristics of patients with missing rapid plasma reagin values and those without missing values at 6 months of follow-up. (DOCX) [file pone.0109667.s005.docx]

| **Table S1.** Comparisons of clinical characteristics of patients with missing rapid plasma reagin values and those without missing values at 6 months of follow-up | | | | | | | | | | | | | | | |
| --- | --- | --- | --- | --- | --- | --- | --- | --- | --- | --- | --- | --- | --- | --- | --- |
|  | Patients with missing values (n=123) | | | | | | Patients without missing values (n=450) | | | | | | | P-value | |
| Age, mean (SD), years | 32.9 (7.3) | | | | | | 33.2 (8) | | | | | | | 0.58 | |
| Risk, n (%) |  |  |  |  | |  | |  |  |  |  | | | |  |
| MSM | 116 (94.31) | | | | 423 (94.00) | | | | | | | | >0.99 | | |
| non-MSM | 7 (5.69) | | | | 27 (6.00) | | | | | | | |  | | |
| Syphilis stage, n (%) |  |  |  |  | |  | |  |  |  |  | | | |  |
| Primary | 14 (11.38) | | | | 37 (8.22) | | | | | | | 0.29 | | | |
| Secondary | 79 (64.23) | | | | 252 (56.00) | | | | | | | 0.12 | | | |
| Early latent | 30 (24.39) | | | | 161 (35.78) | | | | | | | 0.018 | | | |
| RPR titer, median (IQR) |  |  |  |  | |  | |  |  |  |  | | | |  |
| RPR titer ≧ 1:32 | 99 (80.49) | | | | 379 (84.22) | | | | | | | 0.34 | | | |
| CD4 count, mean (SD), cells/μl | 447 (247) | | | | 459 (243) | | | | | | | 0.83 | | | |
| CD4 ≦200, n (%) | 15 (12.20) | | | | 56 (12.44) | | | | | | | >0.99 | | | |
| 200 <CD4 ≦350, n (%) | 33 (26.83) | | | | 103 (22.89) | | | | | | | 0.4 | | | |
| CD4 >350, n (%) | 75 (60.98) | | | | 291 (64.67) | | | | | | | 0.46 | | | |
| PVL, mean (SD), log_10_ copies/ml | 3.15 (1.48) | | | | 3.02 (1.5) | | | | | | | 0.66 | | | |
| PVL <400 copies/ml, n (%) | 62 (50.41) | | | | 243 (54.00) | | | | | | | 0.54 | | | |
| Prior history of syphilis, n (%) | 42 (34.15) | | | | 161 (35.78) | | | | | | | 0.83 | | | |
| CART, n (%) | 78 (63.41) | | | | 284 (63.11) | | | | | | | >0.99 | | | |
| 3 doses of penicillin, n (%) | 65 (52.85) | | | | 213 (47.33) | | | | | | | 0.31 | | | |

**Abbreviations:** CART; combination antiretroviral therapy; IQR, interquartile range; MSM, men who have sex with men; PVL, plasma HIV RNA load; SD, standard deviation
