# Supplementary material for: One Dose versus Three Weekly Doses of Benzathine Penicillin G for Patients Co-Infected with HIV and Early Syphilis: A Multicenter, Prospective Observational Study
Source: PLoS One. 2014 Oct 6;9(10):e109667. doi: 10.1371/journal.pone.0109667 (PMC4186862; doi:10.1371/journal.pone.0109667)
Supplement: Table S2 — Comparisons of clinical characteristics of patients with missing rapid plasma reagin values and those without missing values at 12 months of follow-up. (DOCX) [file pone.0109667.s006.docx]

| **Table S2.** Comparisons of clinical characteristics of patients with missing rapid plasma reagin values and those without missing values at 12 months of follow-up | | | | | | | | | | | | | |  |  |
| --- | --- | --- | --- | --- | --- | --- | --- | --- | --- | --- | --- | --- | --- | --- | --- |
|  | Patients with missing values (n=101) | | | | | Patients without missing values (n=472) | | | | | | | P-value | | |
| Age, mean (SD), years | 31.9 (7.3) | | | | | 33.4 (7.9) | | | | | | | 0.9 | | |
| Risk, n (%) |  |  |  | |  | |  |  |  | |  |  | | |  |
| MSM | 94 (93.07) | | | 445 (94.28) | | | | | | 0.64 | | | | | |
| non-MSM | 7 (6.93) | | | 27 (5.72) | | | | | |  | | | | | |
| Syphilis stage, n (%) |  |  |  | |  | |  |  |  | |  |  | | |  |
| Primary | 14 (13.86) | | | 37 (7.84) | | | | | | 0.08 | | | | | |
| Secondary | 57 (56.44) | | | 274 (58.05) | | | | | | 0.82 | | | | | |
| Early latent | 30 (29.70) | | | 161 (34.11) | | | | | | 0.42 | | | | | |
| RPR titer, median (IQR) |  |  |  | |  | |  |  |  | |  |  | | |  |
| RPR titer ≧ 1:32 | 77 (76.24) | | | 472 (100.00) | | | | | | 0.04 | | | | | |
| CD4 count, mean (SD), cells/μl | 441 (229) | | | 460 (247) | | | | | | 0.193 | | | | | |
| CD4 ≦200, n (%) | 11 (10.89) | | | 60 (12.71) | | | | | | 0.74 | | | | | |
| 200 <CD4 ≦350, n (%) | 24 (23.76) | | | 112 (23.73) | | | | | | >0.99 | | | | | |
| CD4 >350, n (%) | 66 (65.35) | | | 300 (63.56) | | | | | | 0.82 | | | | | |
| PVL, mean (SD), log_10_ copies/ml | 3.29 (1.49) | | | 2.99 (1.49) | | | | | | 0.95 | | | | | |
| PVL <400 copies/ml, n (%) | 47 (46.53) | | | 258 (54.66) | | | | | | 0.15 | | | | | |
| Prior history of syphilis, n (%) | 23 (22.77) | | | 180 (38.14) | | | | | | 0.004 | | | | | |
| CART, n (%) | 48 (47.52) | | | 314 (66.53) | | | | | | <0.001 | | | | | |
| 3 doses of penicillin, n (%) | 56 (55.45) | | | 222 (47.03) | | | | | | 0.13 | | | | | |

**Abbreviations:** CART, combination antiretroviral therapy; IQR, interquartile range; MSM, men who have sex with men; PVL, plasma HIV RNA load; SD, standard deviation
