# Supplementary material for: One Dose versus Three Weekly Doses of Benzathine Penicillin G for Patients Co-Infected with HIV and Early Syphilis: A Multicenter, Prospective Observational Study
Source: PLoS One. 2014 Oct 6;9(10):e109667. doi: 10.1371/journal.pone.0109667 (PMC4186862; doi:10.1371/journal.pone.0109667)
Supplement: Table S3 — Factors associated with serological response in the patients who received 1 dose or 3 doses of benzathine penicillin G at 6 months of follow-up in univariate and multivariate analysis. (DOC) [file pone.0109667.s007.doc]

**Table S3.** Factors associated with serological response in the patients who received 1 dose or 3 doses of benzathine penicillin G at 6 months of follow-up in univariate and multivariate analysis

|  | Univariate analysis | | | Multivariate analysis | | |
| --- | --- | --- | --- | --- | --- | --- |
|  | Responders (n=410) | non-responders (n=163) | P-value | Adjusted odds ratio | 95% confidence interval | P-value |
| Age, mean (SD), years | 32.6 (7.78) | 34.5 (7.75) | 0.92 | 0.97 | 0.95-0.99 | 0.013 |
| Risk, n (%) |  |  |  |  |  |  |
| MSM | 390 (95.1) | 149 (91.4) | 0.12 | 1.64 | 0.77-3.50 | 0.2 |
| non-MSM | 20 (4.9) | 14 (8.6) |  | 1 |  |  |
| Syphilis stage, n (%) |  |  |  |  |  |  |
| Primary | 32(7.8) | 19 (11.7) | 0.15 | 1 |  |  |
| Secondary | 246 (60) | 85(52.1) | 0.09 | 1.38 | 0.83-2.30 | 0.22 |
| Early latent | 132 (32.2) | 59 (36.2) | 0.38 | 1.41 | 0.80-2.49 | 0.23 |
| RPR titer, median (IQR) | 1:64 (32, 128) | 1:64 (32, 128) |  |  |  |  |
| RPR titer ≧ 1:32 | 360 (87.8) | 118 (72.4) | <0.001 | 2.59 | 1.63-4.12 | <0.001 |
| CD4 count, mean (SD), cells/μl | 467 (235) | 431 (264) | 0.15 |  |  |  |
| CD4 ≦200, n (%) | 44 (10.7) | 27 (16.6) | 0.07 | 1 |  |  |
| 200 <CD4 ≦350, n (%) | 92 (22.4) | 44(27.0) | 0.28 | 1.38 | 0.75-2.55 | 0.31 |
| CD4 >350, n (%) | 274 (66.8) | 92 (56.4) | 0.02 | 1.95 | 1.12-3.39 | 0.017 |
| PVL, mean (SD), log_10_ copies/ml | 3.06 (1.50) | 3.02 (1.50) | 0.81 |  |  |  |
| PVL <400 copies/ml, n (%) | 216 (52.7) | 89 (54.6) | 0.71 | 0.87 | 0.47-1.62 | 0.66 |
| Prior history of syphilis, n (%) | 141(34.4) | 62(38.0) | 0.44 | 0.79 | 0.52-1.18 | 0.24 |
| CART, n (%) | 254 (62.0) | 108 (66.3) | 0.34 | 0.85 | 0.56-1.29 | 0.44 |
| 3 doses of penicillin, n (%) | 193 (47.1) | 85(52.1) | 0.31 | 0.88 | 0.60-1.30 | 0.53 |

**Abbreviations:** CART, combination antiretroviral therapy; IQR, interquatile range; PVL, plasma HIV RNA load; RPR, rapid plasma reagin; SD, standard deviation
